# Current data on IL-17 and Th17 cells and implications for graft *versus* host disease

**DOI:** 10.1590/S1679-45082013000200019

**Published:** 2013

**Authors:** Marília Normanton, Luciana Cavalheiro Marti

**Affiliations:** 1 Hospital Israelita Albert Einstein, São Paulo, SP, Brazil; 2 Postgraduate Program in Allergy and Immunology e Imunologia da Faculdade de Medicina, Universidade de São Paulo, São Paulo, SP, Brazil

**Keywords:** IL-17, Th17, Graft-host disease

## Abstract

Human interleukin 17 was first described in 1995 as a new cytokine produced primarily by activated T CD4+ cells that stimulate the secretion of IL-6 and IL-8 by human fibroblasts, besides increasing the expression of ICAM-1. Various authors have reported that IL-17A has a role in the protection of organisms against extracellular bacteria and fungi due to the capacity of IL-17A to recruit neutrophils to the areas of infection, evidencing a pathological role in various models of autoimmune diseases, such as experimental autoimmune encephalitis and arthritis. The participation of IL-17A has also been described in the acute rejection of organ transplants and graft *versus* host disease. However, the greatest revolution in research with IL-17 happened in 2000, when it was proposed that IL-17 cannot be classified as Th1 or Th2, but rather, simply as a new lineage of IL-17-producing T-cells. These findings modified the previously established Th1/Th2 paradigm, leading to the definition of the CD3+ CD4+ Th17 cellular subtype and establishment of a new model to explain the origin of various immune events, as well as its implication in the graft *versus* host disease that is discussed in depth in this article.

## RESUMO

A interleucina 17 humana foi descrita pela primeira vez em 1995, como uma nova citocina produzida principalmente por células T CD4+ ativadas, que estimulam a secreção de IL-6 e IL-8 por fibroblastos humanos, além de aumentar a expressão de ICAM-1. Vários autores relataram que a IL-17A tem um papel na proteção de organismos contra bactérias extracelulares e fungos devido à capacidade de recrutar neutrófilos para as áreas de infecção, evidenciando um papel patológico em vários modelos de doenças autoimunes, como a encefalite autoimune experimental e artrite. A participação da IL-17A também foi descrita na rejeição aguda em transplantes de órgãos e doença enxerto contra hospedeiro. Entretanto, a maior revolução na pesquisa com IL-17 aconteceu em 2000, quando foi proposto que IL-17 não pode ser classificada como Th1 ou Th2, mas sim como uma nova linhagem de células T produtoras de IL-17. Estes achados modificam o paradigma Th1/Th2 previamente estabelecido, levando à definição do subtipo celular CD3+, CD4+ Th17 e ao estabelecimento de um novo modelo para explicar a origem de vários eventos imunes, como também suas implicações na doença enxerto contra hospedeiro, que são bem discutidas neste artigo.

## INTRODUCTION

Graft *versus* host disease (GVHD) is an important clinical complication after hematopoietic stem cell transplantation that can occur acutely within 100 days after bone marrow transplantation, or later as chronic GVHD. Acute GVHD generally affects the skin, liver, and intestinal tract, whereas in its chronic form, the disease can extend to the lung, eyes, and mucous membranes^(1)^.

GVHD initially develops because donor T-cells firstly recognize host alloantigens and become activated. Among the cells involved in GVHD, T helper 1 (Th1) cells are considered the main triggers of the process. These are interferon gamma (IFNγ)-secreting cells that express the T-box transcription factor (T-bet). However, experimental models of GVHD have shown that elimination of Th1 cell activity does not suppress the development of the disease. The description of the cytokine interleukin 17 (IL-17) in 1995 and the subsequent recognition of IL-17-secreting Th cells as a distinct subset named Th17, prompted the investigation of several diseases whose immunopathology could not be totally or partially ascribed to Th1 cells. It was soon determined that IL-17 participated in the process of acute rejection of organ transplantation^(2,3)^.

Thus, the investigation of Th17 cells and IL-17 became especially important concerning GVHD.

In 1995, Yao et al. first described human IL-17, which is mainly produced by activated Th CD4+ cells. It stimulates the secretion of interleukin 6 (IL-6) and interleukin 8 (IL-8) by human fibroblasts and enhances the expression of the intercellular adhesion molecule 1 (ICAM-1)^(3)^.

Subsequently, mouse and human IL-17 receptors (IL-17RA) have been cloned; IL-17RA is considered the receptor for IL-17 and is highly expressed and distinct compared to other cytokine receptors^(4,5)^.

The IL-17 family includes seven me mbers (IL-17 or IL-17A, IL-17B, IL-17C, IL-17D, IL-17E or IL-25, IL-17F, and the viral homologue vIL-17 or ORF13), and nowadays as many as five different receptors have been described^(6)^.

Several reports have proposed that IL-17A has a role in the protection against extracellular bacteria and fungi because of its ability to recruit neutrophils to infected areas. However, it soon became evident that IL-17 participates in the pathology of several autoimmune models of disease, such as experimental autoimmune encephalomyelitis (EAE) and arthritis^(7–10)^.

**Figure 1:**
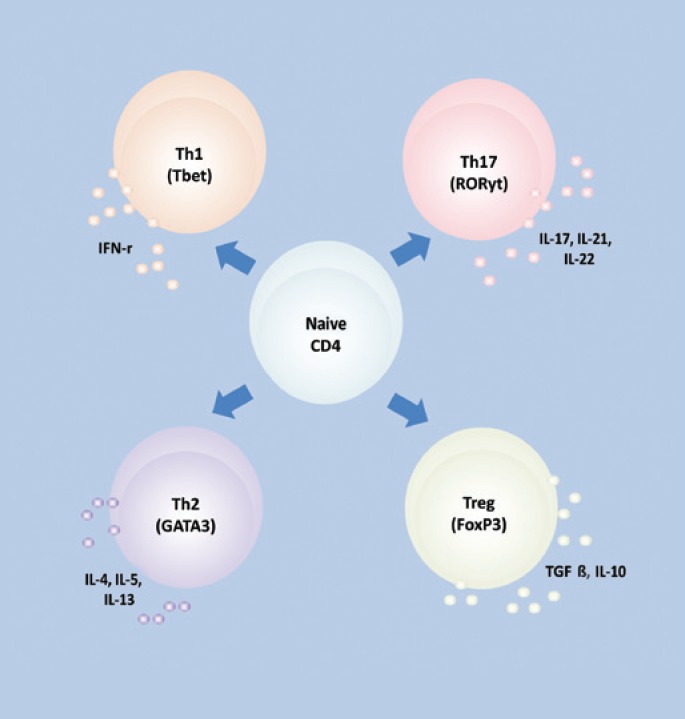
Naive CD4 T-cells can differentiate into diverse phenotypes by cytokines present in the microenvironment where they proliferate. Signaling by cytokines leads to the expression of their transcription factors (Tbet for Th1, RORγt for Th17, GATA3 for Th2, and FOX P3 for Treg) and the synthesis of their so-called signature cytokines. Adapted from Teshima, 2011^(33)^

Nevertheless, the major revolution in IL-17 research occurred in 2000 when Infante-Duarte et al. proposed that IL-17 should not be classified as a Th1 or Th2-derived cytokine, but as a novel T-cell lineage producing IL-17A, as is proposed in figure 1. This concept modified the established Th1-Th2 paradigm leading to the definition of the new Th17 cell subset, and offered new perspectives to the study of several immunological diseases and mechanisms of T-cell regulation. The Th17 cell subset comprises IL-17-secreting cells that express the transcription factor RAR-related orphan receptor gamma (RORγt)^(11,12)^.

Therefore, presently, four basic subsets of differentiated Th cells are recognized, each secreting distinct signature cytokines and expressing characteristic transcription factors, as is summarized in figure 1.

Interleukin-17 and Th17 were first implicated in GVHD physiopathology in 2007^(2)^. This review includes the indexed publications that refer to the relationship of GVHD with Th17 since their first appearance in scientific literature.

## OBJECTIVE

To report the evidence of Th17 and/or IL-17 involvement in the physiopathology of graft *versus* host disease.

## METHODS

This study is a descriptive exploratory narrative review of literature.

Literature search strategies were developed using the medical subject titles “Th17 cells” and “graft *versus* host disease” and all MeSH correlated terms as described on chart 1. Thus, these terms were used to correlate Th17 with GVHD.

**Chart 1:** MeSH guiding questions

MeSH terms	And	MeSH terms
Th17 cells		Graft vs. Host Disease
OR all fields Cell, Th17		OR all fields Graft-Versus-Host Disease
OR all fields Cells, Th17		OR all fields Disease, Graft-Versus-Host
OR all fields Th17 cell		OR all fields Diseases, Graft-Versus-Host
Search “Th17 cell” [MeSH Terms]) OR (Cell, Th17)		OR all fields Graft Versus Host Disease
OR (Cells, Th17) OR (Th17 cell)		OR all fields Graft-Versus-Host Diseases
		OR all fields Graft-vs-Host Disease
		OR all fields Disease, Graft-vs.-Host
		OR all fields Diseases, Graft-vs.-Host
		OR all fields Graft-vs-Host Diseases
		Search “graft vs host disease” [MeSH Terms]) OR (Graft-Versus-Host Disease) OR (Disease, Graft-Versus-Host)
		OR (Diseases, Graft-Versus-Host) OR (Graft Versus Host Disease) OR (Graft-Versus-Host Diseases)
		OR (Graft-vs-Host Disease) OR (Disease, Graft-vs-Host) OR Diseases, Graft-vs-Host) OR (Graft-vs-Host Diseases).

The database was consulted during May 2012 at Medline using the National Center for Biotechnology Information (NCBI) PubMed interface.

We found scientific studies published from 2007 to 2012. This search retrieved 47 articles, and 19 were omitted according to the exclusion criteria shown on chart 2. We found 28 relevant articles.

**Chart 2:** Exclusion criteria chart

N	Article	Exclusion criteria
1	Novel role for surfactant protein A in gastrointestinal graft-versus-host disease. Gowdy KM, Cardona DM, Nugent JL, Giamberardino C, Thomas JM, Mukherjee S, Martinu T, Foster WM, Plevy SE, Pastva AM, Wright JR, Palmer SM.J Immunol. 2012 May 15;188(10):4897-905.	Not related to the Th17 topic
2	Immune modulation of inflammatory conditions: regulatory T-cells for treatment of GvHD. Haase D, Starke M, Puan KJ, Lai TS, Rotzschke O. Immunol Res. 2012 Mar 15.	Not related to the Th17 topic
3	An absence of donor TH17 differentiation ameliorates dermal tissue damage. Cheng H, Song GL, Pan B, Tian J, Yan ZL, Chen W, Xu KL, Li Z Y, Zeng LY. Zhonghua Yi Xue Za Zhi. 2011 Jul 12;91(26):1843-6. Chinese.	Article in Chinese
4	Human TH17 cells are long-lived effector memory cells. Kryczek I, Zhao E, Liu Y, Wang Y, Vatan L, Szeliga W, Moyer J, Klimczak A, Lange A, Zou W. Sci Transl Med. 2011 Oct 12;3(104):104ra100	Not related to the GVHD topic
5	Future perspectives: should Th17 cells be considered as a possible therapeutic target in acute myeloid leukemia patients receiving allogeneic stem cell transplantation? Ersvær E, Melve GK, Bruserud O. Cancer Immunol Immunother. 2011 Dec;60(12):1669-81. Epub 2011 Oct 12. Review.	Not related to the GVHD topic
6	Diminished regulatory T-cells in cutaneous lesions of thymoma-associated multi-organ autoimmunity: a newly described paraneoplastic autoimmune disorder with fatal clinical course. Hanafusa T, Azukizawa H, Kitaba S, Murota H, Umegaki N, Terao M, Sano S, Nakagiri T, Okumura M, Katayama I. Clin Exp Immunol. 2011 Nov;166(2):164-70.	Not related to the GVHD topic
7	Anti-IL-6-receptor-alpha (tocilizumab) does not inhibit human monocyte-derived dendritic cell maturation or alloreactive T-cell responses. Betts BC, St Angelo ET, Kennedy M, Young JW. Blood. 2011 Nov 10;118(19):5340-3.	Not related to the Th17 topic
8	Mesenchymal stem cell effects on T-cell effector pathways. Duffy MM, Ritter T, Ceredig R, Griffin MD. Stem Cell Res Ther. 2011 Aug 11;2(4):34.	Not related to the Th17 topic
9	IDO in human gut graft-versus-host disease. Ratajczak P, Janin A, Peffault de Larour R, Koch L, Roche B, Munn D, Blazar BR, Socié G. Biol Blood Marrow Transplant. 2012 Jan;18(1):150-5	Not related to the Th17 topic
10	The role of Th17 cells in early onset of mice acute graft versus host disease. Cheng H, Zeng LY, Pan B, Song GL, Tian J, Chen C, Yan ZL, Li Z Y, Xu KL. Zhonghua Xue Ye Xue Za Zhi. 2011 May;32(5):322-5. Chinese.	Article in Chinese
11	Detection of Th17/treg cell-associated cytokines in peripheral blood of patients with graft-versus-host disease and its clinical significance. Wang J, Wang XB, Wang J, Liu HL, Geng LQ, Ding KY, Sun ZM. Zhongguo Shi Yan Xue Ye Xue Za Zhi. 2011 Apr;19(2):422-6. Chinese.	Article in Chinese
12	Innate immune activation potentiates alloimmune lung disease independent of chemokine (C-X-C motif) receptor 3. Martinu T, Kinnier C V, Gowdy KM, Kelly FL, Snyder LD, Jiang D, Foster WM, Garantziotis S, Belperio JA, Noble PW, Palmer SM. J Heart Lung Transplant. 2011 Jun;30(6):717-25.	Not related to the Th17 topic
13	Host-microbe interactions in stem cell transplantation: recognizing Candida in infection and inflammation. van der Velden WJ, Plantinga TS, Donnelly J P, Kullberg BJ, Blijlevens NM, Netea MG. Virulence. 2010 May-Jun;1(3):180-4.	Not related to the GVHD topic
14	CD3 mAb treatment ameliorated the severity of the cGVHD-induced lupus nephritis in mice by up-regulation of Foxp3+ regulatory T-cells in the target tissue: kidney. Zhang JL, Sun DJ, Hou CM, Wei YL, Li XY, Yu ZY, Feng JN, Shen B F, Li Y, Xiao H. Transpl Immunol. 2010 Oct;24(1):17-25. Epub 2010 Sep 17.	Not related to the Th17 topic
15	Cbl-b(-/-) T-cells demonstrate in vivo resistance to regulatory T-cells but a context-dependent resistance to TGF-beta. Adams CO, Housley WJ, Bhowmick S, Cone RE, Rajan T V, Forouhar F, Clark RB. J Immunol. 2010 Aug 15;185(4):2051-8.	Not related to the Th17 topic
16	The incidence of acute graft-versus-host disease increases with Candida colonization depending on the dectin-1 gene status. van der Velden WJ, Plantinga TS, Feuth T, Donnelly J P, Netea MG, Blijlevens NM. Clin Immunol. 2010 Aug;136(2):302-6. Epub 2010 May 10.	Not related to the Th17 topic
17	G-CSF induces a potentially tolerant gene and immunophenotype profile in T-cells in vivo. Toh HC, Sun L, Soe Y, Wu Y, Phoon Y P, Chia WK, Wu J, Wong KY, Tan P. Clin Immunol. 2009 Jul;132(1):83-92.	Not related to the GVHD topic
18	Identification of IL-18 and Th17 cells in salivary glands of patients with Sjögren's syndrome, and amplification of IL-17-mediated secretion of inflammatory cytokines from salivary gland cells by IL-18. Sakai A, Sugawara Y, Kuroishi T, Sasano T, Sugawara S. J Immunol. 2008 Aug 15;181(4):2898-906.	Not related to the GVHD topic
19	Pathobiology of transforming growth factor beta in cancer, fibrosis, and immunologic disease, and therapeutic considerations. Prud'homme GJ. Lab Invest. 2007 Nov;87(11):1077-91. Epub 2007 Aug 27. Review.	Not related to the GVHD topic

## RESULTS

Twenty-eight articles on IL-17 related to GVHD were found in our search and chart 3 summarizes each article database, authors, publication year, study type, main theme, and country of origin.

**Chart 3:** Selected articles

N	Database	Title	Authors	Year	Origin	Type of study	Main themes
13	PubMed	Absence of regulatory T-cell control of TH1 and TH17 cells is responsible for the autoimmune-mediated pathology in chronic graft-versus-host disease.	Chen X, Vodanovic-Jankovic S, Johnson B, Keller M, Komorowski R, Drobyski WR.	2007	USA	Experimental	Treg, Th17 cell and chronic GVHD
14	PubMed	Interferon-gamma regulates idiopathic pneumonia syndrome, a Th17+CD4+ T-cell-mediated graft-versus-host disease.	Mauermann N, Burian J, von Garnier C, Dirnhofer S, Germano D, Schuett C, Tamm M, Bingisser R, Eriksson U, Hunziker L.	2008	USA	Experimental	Th17 cell, CD4 T-cell, idiopathic pneumonia syndrome and GVHD
15	PubMed	Absence of donor Th17 leads to augmented Th1 differentiation and exacerbated acute graft-versus-host disease.	Yi T, Zhao D, Lin CL, Zhang C, Chen Y, Todorov I, LeBon T, Kandeel F, Forman, S, Zeng D.	2008	USA	Experimental	Th17 cell, Th1 cell, acute GVHD
16	PubMed	IL-17 contributes to CD4-mediated graft-versus-host disease.	Kappel LW, Goldberg GL, King CG, Suh DY, Smith OM, Ligh C, Holland AM, Grubin, J, Mark NM, Liu C, Iwakura Y, Heller G, van den Brink MR.	2009	USA	Experimental	IL-17, GVHD
17	PubMed	In vitro-differentiated TH17 cells mediate lethal acute graft-versus-host disease with severe cutaneous and pulmonary pathologic manifestations.	Carlson MJ, West ML, Coghill JM, Panoskaltsis-Mortari A, Blazar BR, Serody JS.	2009	USA	Experimental	Th17 cell, GVHD, cutaneous and pulmonary manifestations.
18	PubMed	Reciprocal differentiation and tissue-specific pathogenesis of Th1, Th2, and Th17 cells in graft-versus-host disease.	Yi T, Chen Y, Wang L, Du G, Huang D, Zhao D, Johnston H, Young J, Todorov I, Umetsu DT, Chen L, Iwakura Y, Kandeel F, Forman S, Zeng D.	2009	USA	Experimental	Th1 cell, Th2 cell and Th17 cell, GVHD, tissue association
19	PubMed	IL-21 blockade reduces graft-versus-host disease mortality by supporting inducible T regulatory cell generation.	Bucher C, Koch L, Vogtenhuber C, Goren E, Munger M, Panoskaltsis-Mortari A, Sivakumar P, Blazar BR.	2009	USA	Experimental	IL-21, GVHD, Treg
20	PubMed	Absence of IL-23p19 in donor allogeneic cells reduces mortality from acute GVHD.	Thompson JS, Chu Y, Glass J F, Brown SA.	2010	USA	Experimental	IL-23p19, mortality, GVHD
21	PubMed	T-helper 17 cells are sufficient but not necessary to induce acute graft-versus-host disease	Iclozan C, Yu Y, Liu C, Liang Y, Yi T, Anasetti C, Yu XZ	2010	USA	Experimental	Th17 cell, GVHD
22	PubMed	STAT3 signaling in CD4+ T-cells is critical for the pathogenesis of chronic sclerodermatous graft-versus-host disease in a murine model.	Radojcic V, Pletneva MA, Yen HR, Ivcevic S, Panoskaltsis-Mortari A, Gilliam AC, Drake CG, Blazar BR, Luznik L.	2010	USA	Experimental	Stat3, Th17 cell, GVHD
23	PubMed	Interleukin-17-producing cells increase among CD4+ lymphocytes before overt manifestation of acute graft-versus-host disease.	Dlubek D, Turlej E, Sedzimirska M, Lange J, Lange A	2010	Poland	Clinical	IL-17, GVHD
24	PubMed	Cutaneous GVHD is associated with the expansion of tissue-localized Th1 and not Th17 cells.	Broady R, Yu J, Chow V, Tantiworawit A, Kang C, Berg K, Martinka M, Ghoreishi M, Dutz J, Levings MK.	2010	Canada	Clinical	Th1 cell, Th17 cell, GVHD, cutaneous
25	PubMed	Development of a T(H)17 immune response during the induction of murine syngeneic graft-versus-host disease.	Brandon JA, Jennings CD, Kaplan AM, Bryson JS.	2010	USA	Experimental	IL-17, CD4+ T-cells, transplantation
26	PubMed	Homing in on acute graft vs. host disease: tissue-specific T-regulatory e Th17 cells.	Engelhardt BG, Crowe JE	2010	USA	Review	Th17, GVHD, Treg
27	PubMed	New perspectives on the biology of acute GVHD.	Paczesny S, Hanauer D, Sun Y, Reddy P.	2010	USA	Review	GVHD, allogeneic, transplantation, bone marrow
28	PubMed	Th17/Treg ratio in human graft-versus-host disease.	Ratajczak P, Janin A, Peffault de Latour R, Leboeuf C, Desveaux A, Keyvanfar K, Robin M, Clave E, Douay C, Quinquenel A, Pichereau C, Bertheau P, Mary JY, Socié G	2011	France	Clinical	Th17 cell, Treg, GVHD, gastrointestinal
29	PubMed	IL-17-producing T-cells contribute to acute GVHD in patients undergoing to non-manipulated blood and marrow transplantation.	Zhao XY, Xu LL, Lu S Y, Huang XJ.	2011	China	Clinical	IL-17, Th17 cells, acute GVHD
30	PubMed	Sequential activation of inflammatory signaling pathways during graft-versus-host disease (GVHD): early role for STAT1 and STAT3. Cell Immunol. 2011;268(1):37-46.	Ma HH, Ziegler J, Li C, Sepulveda A, Bedeir A, Grandis J, Lentzsch S, Mapara MY.	2011	USA	Experimental	Stat1, Stat3, GVHD, inflammation
31	PubMed	Blockade of IL-6-signaling inhibits the pathogenesis of CD4+ T-cell-mediated lethal graft-versus-host reaction against minor histocompatibility antigen.	Noguchi D, Wakita D, Ohkuri T, Tajima M, Chamoto K, Kitamura H, Nishimura T.	2011	Japan	Experimental	IL-6, CD4, GVHD,
32	PubMed	Prevention of GVHD while sparing GVL effect by targeting Th1 and Th17 transcription factor T-bet and RORγt in mice.	Yu Y, Wang D, Liu C, Kaosaard K, Semple K, Anasetti C, Yu XZ.	2011	USA	Experimental	GVL, GVHD, Th17 cell, Tbet
33	PubMed	Th1 and Th17 join forces for acute GVHD	Teshima T	2011	Japan	Review	Th1 cell, Th17 cell, GVHD
34	PubMed	Regulatory T-cells and IL-17-producing cells in graft-versus-host disease.	Teshima T, Maeda Y, Ozaki K.	2011	Japan	Review	Treg, IL-17 cell, GVHD
35	PubMed	Abrogation of donor T-cell IL-21 signaling leads to tissue-specific modulation of immunity and separation of GVHD from GVL.	Hanash AM, Kappel LW, Yim NL, Nejat RA, Goldberg GL, Smith OM, Rao UK, Dykstra L, Na IK, Holland AM, Dudakov JA, Liu C, Murphy G F, Leonard WJ, Heller G, van den Brink MR.	2011	USA	Experimental	IL-21, GVHD, GVL
36	PubMed	Amelioration of acute graft-versus-host disease by adoptive transfer of ex vivo expanded human cord blood CD4+CD25+ FoxP3+ regulatory T-cells is associated with the polarization of Treg/Th17 balance in a mouse model	Yang J, Fan H, Hao J, Ren Y, Chen L, Li G, Xie R, Yang Y, Qian K, Liu M	2012	China	Experimental	Treg, Th17 cell
37	PubMed	Protective role of T-bet and Th1 cytokines in pulmonary graft-versus-host disease and peribronchiolar fibrosis	Gowdy KM, Nugent JL, Martinu T, Potts E, Snyder LD, Foster WM, Palmer SM.	2012	USA	Experimental	Th1 cell, Tbet, GVHD
38	PubMed	Synthetic retinoid Am80 ameliorates chronic graft-versus-host disease by down-regulating Th1 and Th17.	Nishimori H, Maeda Y, Teshima T, Sugiyama H, Kobayashi K, Yamasuji Y, Kadohisa S, Uryu H, Takeuchi K, Tanaka T, Yoshino T, Iwakura Y, Tanimoto M.	2012	Japan	Experimental	Chronic GVHD, Th1 cell, Th17 cell
39	PubMed	Altered balance between Th1 and Th17 cells in circulation is an indicator for the severity of murine acute GVHD.	Pan B, Zeng L, Cheng H, Song G, Chen C, Zhang Y, Li Z, Xu K.	2012	China	Experimental	Th1 cell, Th17 cell, GVHD
40	PubMed	The IL-17 differentiation pathway and its role in transplant outcome.	Serody JS, Hill GR.	2012	USA	Review	Acute, chronic GVHD, Th1 cell, Th17 cell

Among these articles, 19 were original research reports carried out in animal models, 4 were clinical studies and 5 were reviews.

The nineteen articles on experimental GVHD reviewed here did not show uniform results, mainly because of different experimental protocols used to investigate the relationship between Th17 and GVHD. Other reasons that explain discrepancies among the several studies are related to the animal models used, to the inactivation of different genes (knockouts), or to differences in the pre-grafting conditioning regimens. Some of the selected articles made major contributions to the field. For instance, Chen et al., 2007, first discussed the imbalance between pro-inflammatory and regulatory cell populations and their respective phenotype in the development of GVHD. Also of note, Carlson et al., in 2008, obtained the *in vitro* differentiation of Th17 cells, and by infusing these cells into the animals, were able to demonstrate that GVHD developed independently of IFNγ. In addition to these, there are several other articles that will be discussed in detail in the next section.

The four clinical studies described and established correlations between Th17 and IL-17 in human GVHD. Some indirect evidence, such as the correlation between Th17/Treg ratios and disease scores, as well as the association between the number of infused Th17 cells and GVHD occurrence are very important in the clinical setting. The few clinical studies reported on this subject are, of course, justified by the inherent limitations in obtaining adequate samples from the patients and the severity of their disease.

The five reviews on the subject summarize the existing research and critically analyze the results by pointing out the consensus as well as the yet unclear aspects.

## DISCUSSION

The 28 articles were grouped according to the main focus of the reported research:

### (A) Evidence that Th17 cells mediate GVHD in murine models of the disease

**Figure 2:**
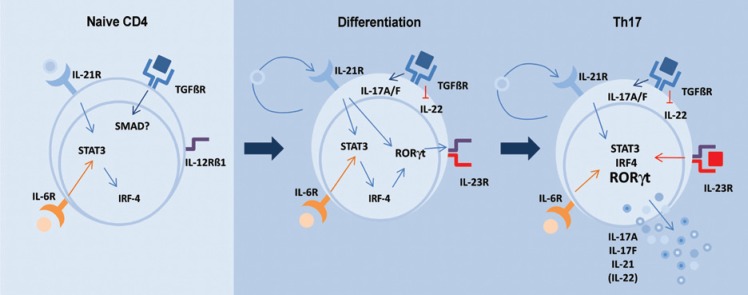
Cytokines IL-21, IL-6, TGFβ acting on the receptors of naive T-cells, activate via STAT 3 the transcription factors IRF-4 and RORγt, leading to the synthesis of IL-17 A and IL-17 F, IL-21, and the expression of the functional IL-23 receptor on the membrane. The synthesis of IL-17 and the transcription factor RORγt are considered the main markers of the Th17 subset. Adapted from Ghilardi and Ouyang, 2007^(12)^

In 2008, Carlson et al. developed a protocol to differentiate naive T-cells into Th17 cells *in vitro*. The main steps and markers of the Th17 differentiation pathway are shown in figure 2. By using purified Th17 cells differentiated from wild-type mice or from mice deprived of the IFNγ genes (IFNγ-/-), the authors showed that Th17 cells can mediate lethal GVHD regardless of IFNγ-production. These cells triggered severe pulmonary and cutaneous manifestations in mice^(17)^. The Th17 competence in inducing GVHD was further confirmed by Iclozan et al., 2010. Furthermore, in the latter study, Th17 cells were identified as more potent than Th1 cells, since small numbers of Th17 cells aggravated the lethality of GVHD in several allogeneic recipients, which demonstrates the pathogenic potential of the Th17 subset^(21)^.

In contrast, also in 2008, Yi et al. showed that the transfer of IL-17-/- donor T-cells to recipient mice exacerbates acute GVHD; worsening of the disease was associated with the expansion of Th1 differentiation and IFNγ production. It was also observed that IL-17 added at low doses to IL-17-/- donor cells reduced the frequency of IFNγ-producing cells, resulting in mitigated acute GVHD^(15)^. In contrast to the preceding articles, this one showed that Th17 cells or IL-17 absence leads to Th1 increase and GVHD exacerbation, and the administration of IL-17 can mitigate GVHD.

In sequence, Kappel et al., 2009^(16)^, demonstrated that donor-derived CD4T-cells after transplantation can produce IL-17 and IFNγ, suggesting that these cytokines are not mutually exclusive and that a single cell is able to produce both of them. They also showed that IL-17 favors GVHD development when purified CD4 T-cells are transferred to allogeneic recipients; however IL-17 is not needed for GVHD development when whole T-cells are transferred to the mice. This article is in accordance with Yi et al. when they state that GVHD can occur in the absence of IL-17, but differs regarding the IL-17 contribution to GVHD, showing that IL-17 favors GVHD instead of protecting against it. However, different experimental protocols used by the authors (for instance, diverse conditioning regimens used before transplantation, and the cell populations used) may have contributed to some of the contrasting results^(15,16)^.

The proposal by Yu et al., 2011, is that naive Th cells can differentiate into Th1 and Th17 and mediate GVHD after adoptive transfer into the allogeneic host. In their experiments they used as donor cells T-cells from mice that had disruptions of both T-bet and RORγt transcription factors, that is, the donor cells were incapable of differentiating into Th1 and Th17 cells. Differentiation occurred instead to Th2 and to T-cells with regulatory phenotypes, resulting in amelioration of established GVHD. They also observed a preserved graft *versus* leukemia (GVL) condition, which is a response against the original disease, usually leukemia cells^(32)^.

An important practical point was raised by Teshima et al., 2011, who made a reference to the paper by Yu et al., 2011, whose results demonstrate that disruption of Tbet and RORγt is beneficial for GVHD without affecting GVL. The question is whether abrogation of Th1/Th17 function, should it be considered as a therapeutic approach, would affect the resistance to opportunistic infections, a recognized major problem after transplantation^(34)^.

In general, all the abovementioned articles, with the exception of that by Yi et al.^(15)^, showed that Th17 cells or IL-17 participate in either triggering or aggravating GVHD.

### (B) Th17/IL-17 in GVHD pathology of selected organs

Because GVHD can affect different organs, several investigators have focused on the role of Th17 in organ-specific manifestations of the disease. This topic includes several articles.

Mauermann et al., 2008, report that the transfer of semi-allogeneic bone marrow in combination with a population of CD4+ T-cells lacking IFNγ or T-bet exacerbated the lung inflammation in recipient mice. In contrast, wild-type donor CD4+ T-cells mediated minimal inflammation, and CD8+ T-cells played a minor role in idiopathic pneumonia syndrome (IPS) development. Furthermore, absence of IFNγ or of IFNγ signaling in the recipients' pulmonary parenchymal cells promoted expansion of IL-17A-producing cells, thus increasing the severity of lung disease. In contrast, IL-17A depletion reduced the disease severity. Perhaps, as discussed by this article, the presence of IL-17A in the lungs promotes neutrophil and macrophage recruitment, aggravating the disease scenario^(14)^. Relative to organ specificity, a few months later, in 2009, Yi et al. published a new article^(18)^ in which they reported the pathogenesis of GVHD affecting specific organs for the activation of specific Th cell subsets. Th1 were found to be involved in gut and liver damage, whereas the absence of IFNγ enhanced lung and skin tissue damage related to Th2 and Th17 cell populations. The authors reported a correlation between Th17 and skin damage, sustaining that Th17 cells are important mediators of skin GVHD because of IL-17 activation of STAT3 in keratinocytes, resulting in epidermal hyperplasia. However they did not observe a significant involvement of Th17 with lung damage as had been reported by Mauerman et al. in their previous article. Yet, Yi et al. consider that Th17 can play a minor role in the pathogenesis of idiopathic pneumonia^(18)^.

In a prior study^(25)^ done before the structure of IL-23 was determined, Brandon et al. reported the inhibition of Th1 in the murine syngeneic GVHD model by the neutralization of the p40 chain of cytokine IL-12. However, now it is known that IL-23 shares the subunit p40 with IL-12. Thus, it is unclear whether therapy with IL-12p40 has blocked Th1 or Th17 development, or both. Therefore, Brandon et al., 2010, reported a study designed to investigate Th17 involvement in syngeneic GVHD-associated colitis (sGVHD). Their experiments demonstrated a significant increase in the number of Th17 cells in sGVHD animals, but the disease score was not modified by administration of anti-IL-17A monoclonal antibodies, leading to the conclusion that IL-12p40 had acted on Th1 instead of Th17 in the previous study, but not excluding a role for Th17/IL-17 in sGVHD^(25)^.

Another approach to test whether pulmonary GVHD can develop in the absence of Th1 cells used T-bet-deficient mice as cell donors in GVHD models. They concluded that pulmonary GVHD development occurs without Th1 cells, and that the absence of T-bet leads to increased production of both Th17 and Th2-type cytokines; the observed lung fibrosis is enhanced by LPS exposure. In addition, LPS-exposed Allo-T-bet(-/-) mice had increased numbers of Th17 cells as well as increased pulmonary IL-17 and IL-13 levels, followed by a reduction in the number of regulatory T-cells^(37)^. Nishimori et al., 2012, investigated Th subsets in chronic GVHD and found up-regulation of Th1, Th2, and Th17 responses. Th1 and Th2 responses were up-regulated first, followed by Th17 cells. Lungs and liver from allogeneic recipients were infiltrated by significantly larger numbers of Th17 cells as compared with the same organs of syngeneic recipients. Reduction of chronic GVHD was seen upon treatment of the mice with AM80, a retinoid that regulates RAR and IL-6, down-regulating both Th1 and Th17. Therefore, in this chronic GVHD model, both these populations and possibly Th2 participate as effector cells, albeit at different time points in the course of the pathogenesis process^(38)^.

### (C) The balance between T regulatory cells and Th1/ Th17 cells in murine models of GVHD

Chen et al., 2007, demonstrated that the autoimmune-like disease that develops as GVHD is attributed to donor-derived CD4 T-cells with Th1 and Th17 cytokine phenotypes. This study importantly describes the unbalanced loss of CD4+ CD25+ Foxp3+ cells, known as T regulatory phenotype cells (Treg), associated with the reciprocal increased secretion of pro-inflammatory cytokines by Th1 and Th17 cells, thus leading to pathological tissue damage in GVHD. This study confirms Th1 and Th17 cells as important to GVHD development, and reinforces the role of Tregs as negative regulators of this disease. In fact Tregs transferred to animals primed to develop GVHD were able to prevent the disease^(13)^.

Engelhardt and Crowe (2010) suggest in their review that Tregs and Th17 cells (but not Th1 cells) are the main T-cell subsets involved in acute GVHD. They hypothesize that both subsets are mutually regulated through retinoic acid, IL-6, and by dendritic cells. Another aspect of the physiology of those T-cell subsets is their homing to lymph node, skin, or gut that is under tight regulation^(26)^.

An excellent review was published by Teshima et al., 2011. They summarize the findings relative to GVHD and Th17 and Treg functions. Based on the accumulated data from experimental and clinical studies, the authors suggest novel future strategies for preventing and treating GVHD, such as Treg cell therapy^(33)^.

Improvement of murine GVHD after adoptive transfer of T human cord blood-expanded Tregs was described by Yang et al., 2012. Their interpretation is similar to that with the Treg/Th17 balance observed previously in the mouse model; the increase in Treg numbers was accompanied by a decrease in Th17 cells. However, the suppressor activity of *in vitro* expanded Tregs and their efficacy in acute GVHD prevention are still poorly understood^(36)^.

Pan et al., 2012, reported findings that also confirmed that an altered balance between Th17 cells and Tregs contributes to several kinds of inflammatory diseases, including acute GVHD^(39)^.

### (D) Role of other cytokines in the Th17 pathway

Interleukin-21 (IL-21) favors Th1 and Th17 differentiation while inhibiting induced regulatory T-cell (iTregs) differentiation. Bucher et al., 2009, demonstrated the IL-21 role in GVHD. Since then, donor T-cell IL-21 production and its IL-21R signaling have been essential for GVHD-induced gastrointestinal tract injury and lethality. IL-21/IL-21R signaling blockage was associated with a decrease in IFNγ- secreting T-cells infiltrating the colon lamina propria, and the consequently diminished disease score. These study data demonstrate that blockade of IL-21 signaling increases iTregs differentiation *in vivo*, but does not abrogate the graft *versus* leukemia (GVL) effect. In addition, they also demonstrated that IL-21 signaling blockage did not affect production of perforin or granzymes, and had no direct effect on CD8 T-cells^(19)^.

IL-23 present heterodimers that share a p40 dimer, but differ by IL-12p35 and IL-23p19. The role of IL-23 to maintain Th17 cells has been shown to be related to its unique dimer p19, and not to the p40 dimer. Thompson et al., 2010, using an allogeneic model of donor p19-/-mouse, have demonstrated that p19 deficiency in allogeneic donor transplantation reduces acute GVHD severity, but increases IL-17 mRNA and serum levels. These results were discussed by the authors, suggesting the effect of p19-/- may be related to interleukin 22 (IL-22), which is another cytokine produced by Th17 cells^(20)^.

Hanash et al., 2011, demonstrated in a murine model of bone marrow transplantation, that IL-21R knockout donor T-cells mediate decreased systemic and gastrointestinal GVHD in transplant recipients. This reduction in GVHD was associated with expansion of donor Treg cells and with tissue-specific modulation of Th cell function^(35)^.

IL-6 signaling blockade by anti-IL-6 receptor monoclonal antibody administration remarkably inhibited lethal GVHD as was demonstrated by Noguchi et al., in 2011. They observed that anti-IL-6R mAb administration did not impair Th1 cells, but cells that simultaneously secrete INFγ, IL-17, and tumor necrosis factor alpha (TNF-α). They also report the increased number of Treg cells in the spleen of treated mice^(31)^.

### (E) Th17 signal transduction role in a murine model of GVHD

To study chronic sclerodermatous GVHD development in animal models, Radojcic et al., 2010, used STAT3 knockout mice to abrogate its signaling. They found that STAT3 absence restricts alloreactive T-cell proliferation and expansion *in vivo*. Although STAT3 abrogation did not impair naive T-cell differentiation into Th1, it promoted natural and induced Treg reconstitution^(22)^.

Sequential activation of signaling pathways during GVHD is the main subject of the article by Ma et al., 2011, which analyzed early STAT1 and STAT3 activation supported by the fact that early STAT3 activation in splenic T-cells is accompanied by IL-17 systemic secretion in GVHD animals. They found that pSTAT1 is upregulated in the early stages of GVHD, not only in spleen cells/lymphocytes but also in the liver and colon; while pSTAT3 is upregulated in early stages and persists during GVHD development. However, further studies will be necessary to functionally dissect the role of STAT1 and STAT3 and their crosstalk during GVHD^(30)^.

### (F) Clinical studies relating Th17 and human GVHD

Dlubek et al., 2010, investigated IL-17-producing cell presence among peripheral blood mononuclear cells (PBMC) from patients after hematopoietic stem cell transplantation (HSCT). All patients presented with an increased number of IL-17-producing cells during hematopoietic reconstitution compared to healthy individuals. However, eight patients developed acute GVHD, displaying lower proportions of IL-17-producing CD4 cells on the day of acute GVHD compared to initial measurements. The author proposes as explanation that these IL-17-producing cells might have migrated to the affected tissues during clinical GVHD manifestations. In this study, however there is no evidence of IL-17 cell presence at the tissue site^(23)^.

In addition, Broady et al., 2010, studied blood and skin from patients with acute GVHD. Even though they found increased IL-17 cells during immune reconstitution, these cells disappeared from circulation in patients with acute GVHD. Nevertheless, the Th17 cells did not migrate to the skin, since they were not increased at GVHD skin sites compared to healthy controls. In contrast, there were significantly more IFNγ-producing T-cells at these skin sites compared to the controls. These data support the long-standing paradigm that tissue IFNγ-producing cells are the main perpetrators of acute GVHD. However, they only investigated the patients' skin, but did not examine other tissues for the presence of Th17 cells^(24)^.

The study by Ratajczak et al., 2011, comprises 96 biopsies in GVHD patients. They explored patients with gastrointestinal and skin GVHD, and the Th17/Treg ratio correlated with both clinical diagnosis and disease severity as assessed by pathologic grade. They concluded that Th17 in gastrointestinal and skin GVHD was not associated with severe tissue damage, but that the Th17/Treg ratio quantification *in situ* was a specific marker of human GVHD. The authors also reported the study limitations, inherent to studies in humans. They had only one time point biopsy from each patient, and it cannot exclude Th17 presence at the beginning of clinical GVHD manifestations^(28)^.

Allografts from forty-one patients were analyzed for IL-17- producing T-cells in acute GVHD onset by Zhao et al., 2011. This group investigated patients who had undergone granulocyte colony-stimulating factor (G-CSF)-mobilized peripheral blood progenitor cells and G-CSF-primed bone marrow transplantation. The results indicate that patients who received higher doses of Th17 cells, G-CSF-primed bone marrow, or a higher dose of Tc17 cells in mobilized peripheral blood progenitor cells exhibited a higher incidence of acute GVHD^(29)^.

Serody and Hill, 2012, conclude in their review that although little is known of IL-17A contribution to GVHD, IL-17A generation is augmented by the use of G-CSF-mobilized grafts, and that this is correlated with disease incidence^(40)^.

Finally, in reviewing literature Paczesny et al., 2010, bring to light the inevitability of robust computational development and mathematical tools to set all emerging experimental data into a multistage model that links intracellular molecular interactions with intercellular behavior to target organ systems^(27)^.

## CONCLUSIONS

Even with controversies, there is evidence in the experimental and clinical studies reviewed here that Th17 is implicated in acute and chronic GVHD physiopathology. Notwithstanding, the detailed Th17 contribution to GVHD or to the Th17/Treg ratio was not totally revealed by these several studies. These questions should be further investigated, which may contribute to the development of new therapies in this field.
